# A mathematical framework for modelling the dynamic nature of ADHD symptoms

**DOI:** 10.3389/fpsyt.2025.1671764

**Published:** 2026-01-09

**Authors:** Marios Adamou, Athanasios Kehagias, Grigoris Antoniou

**Affiliations:** 1 School of Human and Health Sciences, University of Huddersfield, Huddersfield, United Kingdom; 2 Aristotle University of Thessaloniki, Thessaloniki, Greece; 3 School of Built Environment, Engineering and Computing, Leeds Beckett University, Leeds, United Kingdom

**Keywords:** ADHD, delay discounting, hyperactivity, impulsivity, inattention, mathematical modelling, symptom dynamics

## Abstract

**Background:**

Attention-Deficit/Hyperctivity Disorder (ADHD) is characterized by core symptoms of inattention, hyperactivity, and impulsivity that fluctuate dynamically based on context. Standard diagnostic criteria provide static descriptions, failing to capture this variability, while existing computational models may lack interpretability or flexibility for clinical application. There is a need for dynamic, theory-driven models to represent ADHD.

**Objective:**

This study aimed to develop and present a set of interpretable mathematical models representing the dynamic, context-dependent nature of the core symptoms of ADHD, grounded in established neuropsychological principles.

**Methods:**

Algebraic equations were formulated to represent symptom dynamics. Inattention was modelled using modulated exponential decay functions. Hyperactivity was represented by a modulated sinusoidal function reflecting its oscillatory pattern. Impulsive choice was modelled using hyperbolic delay discounting combined with a probabilistic softmax choice rule.

**Results:**

The study produced specific mathematical equations that quantify the temporal dynamics and contextual modulation for each core symptom domain. These equations provide a formal representation of how attention decays, hyperactivity fluctuates, and impulsive choices are made, incorporating individual sensitivities and situational factors pertinent to ADHD.

**Conclusion:**

The proposed mathematical models offer a novel, quantitative framework for understanding and representing the dynamic nature of ADHD symptoms. Grounded in neuropsychological theory, these interpretable models provide a potential advance over static descriptions and may facilitate improved clinical assessment, personalized treatment strategies, and targeted research into the mechanisms underlying ADHD. Further empirical validation is warranted to establish their clinical utility. Further empirical validation is warranted to establish their clinical utility.

## Introduction

Attention-Deficit/Hyperactivity Disorder (ADHD) is a common neurodevelopmental disorder that affects millions of individuals worldwide and contributes substantially to morbidity and long-term disability. The prevalence of ADHD differs between populations and age groups. In children and adolescents, global estimates range from 8% to 10% (1) In adults, two distinct categories are recognized: persistent adult ADHD (symptoms present since childhood) and symptomatic adult ADHD (current symptoms, irrespective of childhood onset). The prevalence of both categories declines with increasing age. After adjustment for the global population structure in 2020, the worldwide prevalence was 2.58% for persistent adult ADHD and 6.76% for symptomatic adult ADHD (2).

ADHD manifests early in childhood, often persisting into adulthood, and is marked by deficits in multiple domains of cognitive and social functioning. ADHD is characterized by pervasive patterns of inattention, hyperactivity, and impulsivity (3). It has complex etiologies that involve a combination of genetic, neurobiological, and environmental factors and presents a significant burden on individuals, families, and healthcare systems. The complexity of ADHD etiology and manifestation has led to an ongoing debate about its diagnosis, treatment, and management strategies. Recent advances in neuroimaging and genetic studies have provided new insights into the underlying mechanisms of ADHD, showing changes in brain structure and function as well as identifying potential genetic markers associated with the disorder. Additionally, the heterogeneity of ADHD symptoms and their overlap with other psychiatric conditions have prompted researchers to explore more personalized approaches to diagnosis and intervention, taking into account individual differences in symptom profiles and comorbidities. However, despite this extensive research, full understanding and quantification of the dynamic nature of this disorder remains a challenge.

ADHD for example manifests through a wide range of symptoms, which often vary considerably between individuals. In ADHD, the core symptoms of inattention, hyperactivity, and impulsivity interact dynamically (4), with symptoms fluctuating based on task demands, environmental stimuli, and internal factors such as arousal or motivation (5). For example, a person with ADHD may have severe inattention during a monotonous task but perform well in a highly stimulating or rewarding environment. Similarly, impulsivity in ADHD is context-dependent, with people showing increased impulsivity in situations where delayed gratification is required. Research has shown that people with ADHD have higher levels of choice impulsivity, particularly in tasks involving delayed rewards. A meta-analysis revealed a medium-magnitude effect size (g=0.47) for choice-impulsivity in children and adolescents with ADHD compared to typically developing peers (6). This indicates that ADHD people tend to prefer smaller immediate rewards over larger delayed ones, demonstrating their difficulty with delayed gratification. The way the symptoms interact with the context complicate diagnosis and make it difficult to predict long-term outcomes or responses to treatment variability.

The current diagnostic frameworks, such as the DSM-5 (3), provide descriptive symptom criteria for ADHD and offer only a static representation of symptoms. The evolving nature of core symptoms over time, particularly in response to external stimuli, is inadequately captured by these static snapshots. In ADHD, symptoms can fluctuate depending on the environment, task demands, and individual context, requiring a more dynamic approach to understanding these conditions. This gap has led to a growing interest in using mathematical modelling to represent the progression, interaction (5), and variability of symptoms over time.

Mathematical models have been applied successfully in various areas of healthcare and mental health to represent complex processes, including disease progression, decision-making, and behavior prediction. In mental health, models based on Bayesian networks, Markov models, and machine learning techniques have been used to classify disorders or predict risk (7–9). Bayesian networks are powerful probabilistic graphical models that represent conditional independence relationships among variables as directed acyclic graphs (DAGs) (10). While they excel at modelling probabilistic relationships and causal effects between variables, traditional Bayesian networks have limitations when it comes to representing continuous symptom variation over time. Recent advances in signal processing and biometrics have also demonstrated the utility of computational frameworks in analyzing physiological states. For instance, studies have utilized dynamic time warping and neural-fuzzy inference systems for authentication and state identification, as well as EEG-driven robust frameworks for signal analysis (11–13).

While Machine Learning models like Support Vector Machines (SVMs) and Convolutional Neural Networks (CNNs) have demonstrated high accuracy in disorder classification, they often suffer from a “black box” nature that limits clinical interpretability (14). Similarly, while Hidden Markov Models (HMMs) are effective for longitudinal data (15, 16), they can lack the flexibility to represent the specific neuropsychological interactions inherent to ADHD.

Given these limitations, there is a need for mathematical models that can directly represent the core symptoms of ADHD and predict their progression over time. Our approach is grounded in cognitive and neuropsychological theory, incorporating the core symptoms of attention, impulsivity, and hyperactivity. By focusing on the core symptoms of ADHD we aim to create a model that captures the temporal dynamics of the symptoms of this disorder. The goal is to provide a framework that can enhance both diagnostic precision and treatment personalization, offering clinicians and researchers a tool to predict symptom trajectories and tailor interventions to individual needs.

### Rationale for a dynamic mathematical model

Mathematical modelling offers a promising approach to addressing the limitations of static diagnostic frameworks by providing a means to represent continuous symptom variation and dynamic interactions between core symptoms. Our model focuses on quantifying the core symptoms of ADHD drawing from well-established neuropsychological research. This mathematical approach allows for a better understanding of ADHD symptomatology, capturing fluctuations and interplay between different symptoms over time. By incorporating dynamic interactions, the model can potentially predict symptom trajectories and identify critical points for intervention. Furthermore, this quantitative framework may facilitate personalized treatment strategies by accounting for individual differences in symptom patterns and severity.

For ADHD, we represent inattention as a function of time, using an exponential decay model to capture the rapid decline in attention during prolonged tasks The model’s representation of inattention as a time-dependent function utilizing an exponential decay model corresponds to observed patterns in people diagnosed with ADHD, who frequently experience difficulties in sustaining focus during prolonged activities.

Hyperactivity is modelled as a sinusoidal function, reflecting the oscillatory nature of restlessness and physical movement, which often occurs in response to environmental stimuli. In ADHD, abnormally low tonic extracellular dopamine levels lead to hypersensitivity to environmental stimuli, resulting in boosted phasic dopamine responses (17). This hypersensitivity can manifest as hyperactivity, especially in environments with impoverished stimuli, as a compensatory mechanism for low arousal (17).

Impulsivity, particularly in decision-making, is modelled using a delay discounting function, representing how people with ADHD devalue delayed rewards in favor of immediate gratification. People with the disorder tend to prefer smaller immediate rewards over larger delayed rewards (18). This preference for immediate gratification is considered a central component of ADHD and has been emphasized in a etiological models of the disorder (19). Studies have shown that people with ADHD, particularly those with the combined subtype (ADHD-CT), discount rewards more steeply than controls in hypothetical tasks (18). Interestingly, the type of task (real vs. hypothetical) and the magnitude of the reward can influence discounting behavior differently in ADHD and control groups. Genetic factors, such as variations in dopamine-related genes (DAT1 and COMT), have been linked to discounting rates and trait impulsivity in both ADHD and healthy individuals (18).

By focusing on these core domains, our mathematical model aims to provide a dynamic representation of how symptoms in ADHD evolve over time and interact with external factors. The model offers potential applications in both clinical diagnostics and treatment planning, allowing for personalized interventions based on individual symptom trajectories.

### Aims of the study

Our aim in this study was to develop a set of mathematical equations that model the dynamic nature of core symptoms in ADHD. By capturing the fluctuating nature of these symptoms, the study intends to show how these models can predict symptom progression and inform personalized treatment strategies. Ultimately, the study seeks to provide a framework for integrating dynamic symptom modelling into clinical practice, with the goal of improving diagnostic accuracy and individualized care. The following sections will detail the mathematical equations developed to represent the core symptoms of ADHD, including their scientific justification and potential applications.

## Methodology

This study employs a mathematical modelling approach to the core symptoms of ADHD. Our goal is to create a dynamic framework that captures the temporal progression and interaction of symptoms based on neuropsychological principles and empirical evidence. By selecting core symptom domains and applying specific equations, we aim to offer a robust, interpretable model for clinicians and researchers to better understand and manage these conditions.

### Symptom selection and rationale

The descriptions of ADHD symptoms are outlined in the DSM-5 (3). The diagnostic criteria for ADHD include 18 symptoms, divided into two categories: inattention (9 symptoms) and hyperactivity/impulsivity (9 symptoms). However, not all symptom descriptors described in the DSM-5 can be mathematically modelled due to their subjective or context-specific nature.

For ADHD, we focused on the core, measurable symptoms of inattention, hyperactivity, and impulsivity. These symptoms were selected because they are central to the disorder’s clinical presentation and have been extensively studied in neuropsychological research, allowing us to draw upon empirical data to guide our modelling choices.

### ADHD: mathematical representations of core symptoms

#### Inattention

Attention is described as the mechanism by which the mind selectively focuses on specific stimuli while ignoring others. William James famously defined it as the “taking possession by the mind, in clear and vivid form, of one out of what seem several simultaneously possible trains of thought.” (20) This selectivity allows for cognitive clarity and efficiency in environments with competing stimuli. Attention can be defined as the selective focus of the mind on specific stimuli, both external and internal, while filtering out other information. The importance of attention lies in its ability to help the brain priorities and process the vast amount of information it receives, enabling effective perception, decision-making, and action.

Attention is often characterized by its selective nature, as the brain cannot fully process all the available sensory information simultaneously. This selectivity can be driven by either exogenous (stimulus-driven) or endogenous (goal-driven) factors, leading to bottom-up or top-down attentional processes, respectively (21). Attention comprises several interrelated components. These include selectivity, which refers to the brain’s ability to focus on relevant information; processing resources, which are the cognitive capacities dedicated to attending to specific stimuli; and arousal, which is the state of physiological and psychological responsiveness. Sustained attention — the ability to maintain focus over an extended period — is also considered an important aspect of attention, along with divided attention. Divided attention refers to the capacity to process multiple streams of information simultaneously or switch rapidly between them. It is often measured through dual-task paradigms, where individuals perform two tasks concurrently, assessing performance on each task as a function of task complexity and resource allocation. Executive control also plays a role in attention, involving the higher-order cognitive functions that regulate and coordinate the various attentional processes (21, 22).

Inattention in ADHD is defined by difficulties in sustaining attention, particularly during tasks that require prolonged mental effort. The DSM-5 lists symptoms such as “fails to give close attention to details’’ and “difficulty sustaining attention in tasks or play activities’’ (3). Studies show that people with ADHD experience a rapid decline in attention over time, especially during monotonous or repetitive tasks. Both ADHD and non-ADHD populations experience attention decline during monotonous tasks, though the mechanisms and severity differ. In ADHD, this decline is more pronounced and linked to deficits in executive functioning and motivation, while in non-ADHD populations, it is primarily due to the depletion of executive resources. Individuals with ADHD and those without both exhibit diminished attention during monotonous tasks, yet the underlying mechanisms and severity of this decline differ. Research indicates that while attentional fatigue in non-ADHD individuals appears primarily due to the depletion of executive resources, the decline is more pronounced in people with ADHD (23). This accentuated decline is associated with deficits in executive functions, such as working memory and inhibitory control, essential for maintaining focus, particularly during repetitive activities (24). Furthermore, motivational factors are implicated, as the reward system in individuals with ADHD may be less responsive, making it more challenging to sustain attention towards tasks perceived as unrewarding (23). Neuroimaging studies corroborate these findings, demonstrating insufficient allocation of neuronal resources and hypoactivation in brain regions responsible for attention and executive function in individuals with ADHD (25, 26). Therefore, the attentional difficulties experienced by people with ADHD during monotonous tasks are not solely attributable to mental fatigue but rather reflect a complex interplay of executive function deficits and motivational challenges, underpinned by distinct neural activation patterns.

To represent this process, we employed an exponential decay model. The decay captures how attentional resources deplete over time, and the steepness of the decay can be adjusted to reflect individual differences in task performance and cognitive endurance.

Our proposed model is grounded in theories of cognitive load and attentional capacity, which suggest that individuals with ADHD experience more rapid depletion of attentional resources compared to neurotypical individuals. The exponential decay function enables the prediction of attention loss over time and offers insights into how to structure tasks for individuals with ADHD, such as including breaks or varying task demands to maintain attentional focus.

#### Hyperactivity

Hyperactivity is characterized by excessive movement and difficulty remaining still, as described in the DSM-5 symptoms: “often fidgets with hands or feet or squirms in seat’’ and “often leaves seat in situations where remaining seated is expected’’ (27).

Hyperactivity in refers to behaviors that exceed what is developmentally appropriate for an individual’s age. These behaviors commonly include excessive motor activity such as fidgeting, tapping hands or feet, an inability to remain seated, and running or climbing in inappropriate settings. Individuals may also struggle with tasks requiring quietness or sustained focus, such as reading or listening. Hyperactivity often manifests as constant movement, with a person appearing as though “driven by a motor,” exhibiting persistent movement or talking. Restlessness is another feature, seen in the tendency to frequently leave a seat in situations where sitting is expected, such as in classrooms or workplaces. Excessive motor activity is perhaps the most recognizable aspect of hyperactivity. This can manifest as fidgeting, and an inability to remain seated in situations where it is expected, such as in classrooms or during meals. Children with hyperactivity may also exhibit a constant need for movement, such as running or climbing in inappropriate situations, or talking excessively. This excessive motor activity can significantly impact their daily functioning, including academic performance and social interactions, as it often interferes with their ability to focus and engage in structured activities The hyperactive behaviors often lead to significant impairment in social, academic, and occupational functioning, as they can disrupt both the individual and those around them (28).

Research indicates that this hyperactive behavior is not merely a symptom of impulsivity but represents a distinct dimension of ADHD that can be assessed independently (29–31). These symptoms seem to be linked to functional and structural neuroimaging studies which consistently implicate a dysfunction in fronto-striatal circuits, including the lateral prefrontal cortex, dorsal anterior cingulate cortex, caudate, and putamen (32). However, abnormalities extend beyond these regions to include areas like the cerebellum and parietal lobes (33). Functional MRI studies have focused on prefrontal and temporal regions, reflecting alterations in perception-action mapping (34).

Interestingly, the pattern of hyperactivity in ADHD does not appear to be strictly linear or cyclical, but rather manifests as a complex interplay among large-scale brain circuits (35). This is evidenced by studies using resting-state approaches to map functional connectivity, which have provided detailed information about interregional relationships (36). The abnormal patterns observed include decreased activations in medial and dorsolateral frontal areas associated with error processing and attention (37), as well as alterations in the posterior cingulate, temporal, and occipital cortex (37).

However, behaviorally, hyperactive behavior follows a cyclical pattern, were periods of intense activity alternate with calmer intervals. This cyclical pattern is characterized by alternating periods of intense activity and calmer intervals, which can be influenced by various factors, including cognitive demands and environmental contexts. Kofler et al. (28) highlights that hyperactive behavior in ADHD is not constant but varies depending on task demands. Their research indicates that individuals with ADHD exhibit significantly less hyperactivity during tasks that require episodic buffer processes compared to those that engage visuospatial working memory. This suggests that hyperactivity may diminish when cognitive resources are allocated to more demanding tasks, supporting the notion of a cyclical pattern where hyperactivity fluctuates based on situational factors. They suggest that hyperactivity in ADHD is not merely a reflection of raw activity levels but is also dependent on environmental demands and task contexts. The findings indicate that hyperactive behaviors can be modulated by the surrounding environment, leading to periods of increased activity followed by intervals of reduced movement. This variability underscores the complexity of hyperactivity as a symptom of ADHD, suggesting that it may not be uniformly present but rather contextually driven. Additionally, research on the neurobiological underpinnings of ADHD supports the cyclical nature of hyperactivity. For instance, studies have shown that dopaminergic activity, which is often heightened in ADHD, can lead to fluctuations in behavior based on reinforcement schedules and environmental stimuli (38). The dynamic developmental theory of ADHD posits that hyperactive behaviors are influenced by alterations in dopaminergic pathways, which can result in varying levels of activity depending on the context and reinforcement mechanisms (38). Moreover, the concept of behavioral variability in ADHD has been explored in the context of reinforcement processes. Johansen et al. found that alterations in reinforcement gradients can lead to significant behavioral variability, including cycles of hyperactivity and calmness (39). This suggests that the behavioral patterns observed in ADHD are not static but are influenced by the interaction between neurobiological factors and environmental conditions.

To model this, we selected a sinusoidal function, which captures the periodic nature of hyperactivity. We acknowledge that human behavior is not perfectly periodic; however, a sinusoidal function serves as a necessary baseline approximation to represent the oscillatory “rest-activity” cycles observed in ADHD, distinct from linear or static models. In real-world application, this function would likely be superimposed with stochastic noise (ϵ) to account for irregular variation.

#### Impulsivity

Impulsivity, at its core, is a behavioral tendency characterized by acting without adequate forethought, reflection, or consideration of the potential consequences. This predisposition often leads to actions that are poorly conceived, prematurely expressed, unduly risky, or inappropriate for the situation, ultimately jeopardizing long-term goals and strategies for success. Impulsivity is a complex construct that has been conceptualized through various models in psychological research. The literature presents several approaches to understanding impulsivity, including trait models, behavioral models, and neuroimaging studies. Trait models of impulsivity have converged towards a three-trait framework, encompassing motivational drive (extraversion), effortful control (conscientiousness/constraint), and emotion dysregulation (neuroticism) (40). The UPPS-P scale, a multidimensional inventory, assesses five personality pathways contributing to impulsive behavior: negative urgency, lack of perseverance, lack of premeditation, sensation seeking, and positive urgency (41). These trait models provide a better understanding of impulsivity as a heterogeneous construct. Interestingly, behavioral models of impulsivity focus on a two-factor model centered around impulsive choice and impulse response, which appears to have little theoretical or empirical connection with trait models (40). This disconnect highlights a significant challenge in integrating trait and behavioral research on impulsivity. Furthermore, some studies suggest a three-factor model of impulsivity, comprising cognitive impulsivity, behavioral impulsivity, and impatience/restlessness (42) while others propose a model based on impulsive choice, impulsive action, and impulsive personality traits (43).

In the context of ADHD, impulsivity is recognized as one of the three core symptom domains, alongside inattention and hyperactivity. DSM-5 provides specific criteria for diagnosing ADHD, which include several symptoms indicative of impulsivity and hyperactivity combined. To meet the diagnostic criteria, individuals must exhibit at least six combined symptoms (for children up to 16 years old) or at least five combined symptoms (for those 17 years and older), with these symptoms having persisted for at least six months and negatively impacting functioning in two or more settings.

The DSM-5 lists the following criteria for impulsive symptoms in ADHD: difficulty waiting their turn, interrupting or intruding on others’ conversations or activities, and impulsively blurting out answers before questions have been completed. For older adolescents and adults, impulsivity might also manifest as taking over what others are doing. Impulsivity in ADHD is characterized by a reduced capacity for behavioral control, where the urge to act arises more rapidly than the cognitive processes that would typically inhibit or modify the action. This highlights the temporal aspect of impulsivity in ADHD – a tendency to respond quickly without adequate processing of the situation.

While impulsivity is a trait that exists on a continuum in the general population, in people with ADHD, it tends to be more frequent, severe, and developmentally inappropriate. The primary distinction lies in the degree to which impulsivity interferes with an individual’s functioning across various aspects of life, such as academic, social, and occupational domains.

Impulsivity is frequently conceptualized through the framework of delay discounting, which denotes the tendency to devalue rewards that are delayed in time in preference for more immediate gratification. In the present context, a delay discounting model was employed to represent this dynamic, whereby the probability of selecting an impulsive option diminishes as the temporal delay to reward receipt increases. This approach reflects the probabilistic character of impulsive behavior, particularly in people with ADHD, who demonstrate a heightened propensity to favor immediate over delayed rewards. Given its extensive application in ADHD research, delay discounting offers a suitable and validated framework for quantifying impulsive decision-making behavior.

## Results

### Mathematical modelling of attention in ADHD

ADHD is characterized by difficulties in sustaining attention, particularly during tasks that require prolonged mental effort. To quantitatively describe these core symptoms, we propose a set of mathematical models that build upon the exponential decay framework, incorporating factors such as task characteristics and motivational influences. These models offer a nuanced representation of the attentional challenges faced by individuals with ADHD, aligning with neuropsychological descriptions of attention as a resource that depletes over time.

#### Sustained attention

Sustained attention, or the ability to maintain focus over prolonged periods, is modeled as decaying exponentially over time. The model is formulated as:


$$A(t)=A_{0}e^{-\beta_{effective}t}$$

Parameters:- A(t): Level of sustained attention at time t.- $A_{0}$: Initial level of sustained attention.- $\beta_{effective}$: Effective decay rate, determined by: $\beta_{effective}=\beta_{base}\frac{1+k_{M}M}{1+k_{R}R}$, $\beta_{base}$: Baseline attentional decay rate, reflecting inherent executive function capacities. - M: Perceived monotony of the task. - $k_{M}$: Sensitivity to monotony, indicating how strongly monotony accelerates attention decline. - R: Perceived reward value or interest level of the task. - $k_{R}$: Sensitivity to reward’s mitigating effect on attention decay.

This fractional formulation represents the opposing forces on attention span. The numerator 1+k_M_M acts as an accelerator of decay, where higher Monotony (M) increases the rate of attention loss. Conversely, the denominator 1+k_R_R acts as a protective factor, where higher Reward (R) or interest reduces the effective decay rate. This aligns with clinical observations that individuals with ADHD can sustain focus in high-reward contexts despite their baseline deficits.

This formulation captures the clinical observation that attention in individuals with ADHD deteriorates more rapidly during monotonous tasks but can be better sustained if the task is perceived as rewarding. The modulated decay rate reflects the dynamic interplay between task characteristics and motivational influences.

#### Attention to detail

Difficulties attending to details are modeled similarly, representing the decay in the precision or specificity of focus:


$$D(t)=D_{0}e^{-\gamma_{effective}t}$$

Parameters:- D(t): Level of attention to detail at time t.- $D_{0}$: Initial level of detail focus.- $\gamma_{effective}$: Effective decay rate for attention to detail, influenced by: $\gamma_{effective}=\gamma_{base}\frac{1+{k^{\prime }}_{M}M}{1+{k^{\prime }}_{R}R}$, - $\gamma_{base}$: Baseline decay rate for attention to detail. - ${k^{\prime }}_{M},{k^{\prime }}_{R}$: Sensitivity coefficients specific to the detail aspect.

This model allows for potentially different baseline decay rates or sensitivities, reflecting the multifaceted nature of ADHD symptoms. It captures how attention to detail can be influenced by task monotony and perceived reward.

#### Effort expenditure

The avoidance of tasks requiring sustained mental effort is linked to a more rapid decline in the capacity or willingness to exert that effort. This is modeled as:


$$E(t)=E_{0}e^{-\lambda_{effective}t}$$

Parameters:- E(t): Level of mental effort being expended at time t.- $E_{0}$: Initial level of effort.- $\lambda_{effective}$: Effective decay rate for effort, determined by: $\lambda_{effective}=\lambda_{base}\frac{1+{{k^{\prime }}^{\prime }}_{M}M}{1+{{k^{\prime }}^{\prime }}_{R}R}$, - $\lambda_{base}$: Baseline rate of effort decline, related to cognitive fatigue tolerance. - ${\lambda^{\prime \prime }}_{M},{\lambda^{\prime \prime }}_{R}$: Sensitivity coefficients capturing how monotony and reward influence the ability to maintain effort.

This model reflects the motivational deficits described in ADHD, where reward sensitivity ( ${k^{\prime \prime }}_{R}$) is particularly relevant. It captures how effort quickly diminishes during demanding cognitive tasks, especially when tasks are perceived as unrewarding.

These mathematical representations utilize a modulated exponential decay framework to model ADHD inattention symptoms. By incorporating parameters for baseline decay rates and sensitivities to task monotony and reward, they provide a quantitative approach to describing how attentional resources and effort decline over time. This nuanced model aligns with the neuropsychological narrative of ADHD, reflecting the specific challenges faced by individuals with the disorder. Future research could explore how these models can inform the development of interventions and task structures that support sustained attention and effort in individuals with ADHD.

### Mathematical representation of hyperactivity in ADHD

Hyperactivity in ADHD is characterized by excessive motor activity that follows a cyclical pattern, with periods of heightened movement alternating with relative calmness.

To mathematically represent this behavior, we propose a refined sinusoidal model that captures the oscillatory nature of hyperactivity while incorporating contextual modulation. The model represents the level of hyperactive behavior (H(t)) at time t as fluctuations around a baseline level, with the amplitude of these fluctuations dynamically adjusted by contextual factors. The equation is given by:

$H(t)=H_{baseline}+H_{effective}\text{sin}\left(\omega t+\varphi \right)$. Here,

$H_{baseline}$ represents the individual"s baseline level of motor activity, and

$H_{baseline}$ is the effective amplitude of the hyperactive fluctuations, determined by:


$$H_{effective}=\frac{H_{\max}}{1+k_{C}C+k_{E}E+k_{R_{C}}R_{C}}$$

where $H_{\max}$ is the potential maximum amplitude of hyperactivity in low-demand contexts, C represents cognitive demand, E represents environmental demands for stillness, and $R_{C}$ represents reinforcement for calm behavior. The coefficients $k_{C},k_{R},k_{R_{C}}$ reflect individual sensitivities to these factors. The angular frequency ω and phase shift φ determine the cyclical pattern of hyperactivity.

We acknowledge that human behavior is not perfectly periodic; however, a sinusoidal function serves as a necessary baseline approximation to represent the oscillatory “rest-activity” cycles observed in ADHD, distinct from linear or static models. In real-world application, this function would likely be superimposed with stochastic noise (ϵ) to account for irregular variations.


$$H\left(t\right)\;=\;H_{baseline}+\;H_{effective}sin\left(\omega t\;+\;\varphi \right)$$

Where:

- H_baseline_: The individual’s baseline level of motor activity.
- ω and φ: Frequency and phase shift determining the cycle.
- H_effective_: The amplitude of the fluctuations, modulated by context:

$$H_{effective}=\frac{H_{\max}}{1+k_{C}C+k_{E}E+k_{RC}R_{C}}$$

In this equation, the amplitude is suppressed by external constraints. Higher Cognitive Demand (C), Environmental restrictions (E), or Reinforcement for Calmness (R_C_) serve to reduce the amplitude of hyperactive outbursts (increasing the denominator). Conversely, in low-demand contexts, the amplitude approaches H_max_.

This model aligns with neuropsychological observations by capturing the dynamic interplay between contextual influences and hyperactive behavior, providing a nuanced representation of real-world behavioral patterns in individuals with ADHD.

### Mathematical representation of impulsivity in ADHD

Impulsivity, particularly in decision-making involving delayed rewards, is effectively modelled using the framework of delay discounting. This approach quantifies the tendency, often heightened in ADHD, to prefer smaller immediate rewards over larger rewards delivered after a delay, reflecting a steeper devaluation of future outcomes. The model involves calculating the subjective value of delayed rewards and then modelling the probabilistic choice between options.

#### Subjective value of delayed rewards

The subjective value ( V) of a reward decreases hyperbolically as the delay ( D) to its receipt increases. The standard formula is:


$$V=\frac{A}{1+kD}$$

Parameters:- V: Subjective value of the reward at the time of decision.- A: Objective amount or magnitude of the reward.- D: Delay until the reward is received.- k: Individual’s discounting rate, quantifying how rapidly the subjective value decreases with delay. A higher k indicates steeper discounting and greater impulsivity in choice tasks. Research suggests individuals with ADHD tend to exhibit higher k values.

#### Probabilistic choice model

When faced with a choice between a Smaller, Sooner Reward (SSR) and a Larger, Later Reward (LLR), the probability of choosing the impulsive option (SSR) can be modeled using the softmax (or logistic) choice rule:


$$P\left(Choose_{SSR}\right)=\frac{1}{1+e^{\beta \left(V_{LLR}-V_{SSR}\right)}}$$

Parameters:- $P\left(Choose_{SSR}\right)$: Probability of making the impulsive choice (selecting the immediate reward).- $V_{LLR}$: Subjective value of the larger, delayed reward, calculated as $\frac{A_{LLR}}{1+kD_{LLR}}$. - $V_{SSR}$: Subjective value of the smaller, immediate reward, calculated as $\frac{A_{SSR}}{1+kD_{SSR}}$ (often $D_{SSR}=0$, simplifying $V_{SSR}$ to $A_{SSR}=0$).- β: Choice consistency parameter, reflecting the sensitivity of choices to the difference in subjective values. Higher β indicates more deterministic choices based on value, while lower β suggests more variability or randomness.

This two-part model aligns well with the neuropsychological theories by:- Using the Hyperbolic Function: The empirically supported hyperbolic function represents the devaluation of delayed rewards, capturing the tendency to prefer immediate gratification.- Identifying the Discounting Rate ( k): This parameter reflects individual differences in impulsive choice, consistent with findings in ADHD.- Providing an Explicit Mechanism: The softmax function translates subjective values into choice probabilities, capturing the probabilistic nature of behavior.

While delay discounting primarily addresses impulsive choice, it provides a robust quantitative framework for understanding one of the core manifestations of impulsivity, particularly relevant to ADHD research and clinical understanding.

## Conclusions

This paper addressed the limitations inherent in static diagnostic frameworks for understanding ADHD, a condition characterized by its dynamic and context-dependent symptom presentation. Standard diagnostic criteria, while essential for diagnosis, inadequately capture fluctuations in inattention, hyperactivity, and impulsivity influenced by environmental demands, cognitive load, and motivational factors. Existing computational approaches, including machine learning and Hidden Markov Models, often lack interpretability or the flexibility required to fully represent the nuanced, moment-to-moment variations observed clinically.

In response, this study proposed a novel approach, developing a set of mathematical equations grounded in established neuropsychological principles to model the core symptoms of ADHD dynamically. For **inattention**, a modulated exponential decay model was formulated, accounting for the accelerated decline in focus during monotonous tasks and the mitigating influence of perceived reward. For **hyperactivity**, a refined sinusoidal model was presented, where the amplitude of behavioral oscillations is modulated by cognitive demand, environmental constraints, and reinforcement contingencies. For **impulsivity**, the framework of delay discounting was employed, using a standard hyperbolic function to determine subjective reward value and a probabilistic choice function (softmax) to model the likelihood of choosing immediate gratification, incorporating the characteristic steeper discounting associated with ADHD.

The primary contribution of this work is the formulation of these specific, theory-driven equations that offer an interpretable, quantitative representation of ADHD symptom dynamics. These models provide a framework that moves beyond static descriptions, enabling a more nuanced understanding of how symptoms evolve over time and interact with external and internal factors. Potential implications include enhancing diagnostic assessment by characterizing individual symptom patterns and sensitivities, facilitating personalized treatment planning by predicting symptom trajectories under different conditions, and providing researchers with quantitative tools to test hypotheses regarding symptom mechanisms and interactions.

Several limitations must be acknowledged. The models presented simplify highly complex neurobehavioral phenomena and focus on specific, measurable facets of the core symptoms (e.g., delay discounting for impulsive choice, not all manifestations of impulsivity). Furthermore, these models require empirical validation using longitudinal data from individuals with ADHD to confirm their accuracy and estimate individual-specific parameters reliably. The explicit mathematical modelling of interactions *between* the different symptom domains also remains an area for future development.

It is important to clarify the scope of this work. While empirical validation and parameter estimation are important next steps, the primary objective of this work is to establish the theoretical mathematical framework and the derivation of equations based on neuropsychological first principles. Providing a robust parameter estimation method and fitting it to clinical data would necessitate a separate, extensive dataset and longitudinal study, which lies outside the scope of this initial theoretical proposition.

Future research should therefore prioritize the empirical testing and refinement of these models. We propose that researchers approach parameter estimation by utilizing Ecological Momentary Assessment (EMA) data to capture real-time symptom fluctuations. Methods such as non-linear least squares fitting or Bayesian inference could then be applied to this data to estimate individual-specific parameters (e.g., β_base_, k__M_, ω).

In conclusion, the dynamic mathematical modelling approach proposed here offers a promising avenue for advancing the scientific understanding and clinical management of ADHD. By providing an interpretable, quantitative framework grounded in neuropsychological theory, these models have the potential to improve assessment, personalize interventions, and ultimately enhance outcomes for individuals affected by this complex neurodevelopmental disorder.

## Data Availability

The raw data supporting the conclusions of this article will be made available by the authors, without undue reservation.
